# The combination of temozolomide and perifosine synergistically inhibit glioblastoma by impeding DNA repair and inducing apoptosis

**DOI:** 10.1038/s41420-024-02085-1

**Published:** 2024-07-08

**Authors:** Wenpeng Zhao, Liwei Zhou, Wentao Zhao, Huiying Yang, Zhenwei Lu, Liang Zhang, Yaya Zhang, Yuanyuan Xie, Hanwen Lu, Wanhong Han, Jiawei He, Xiansheng Qiu, Fang Jia, Wujie Zhao, Bingchang Zhang, Zhanxiang Wang

**Affiliations:** 1 Department of Neurosurgery and Department of Neuroscience, Fujian Key Laboratory of Brain Tumors Diagnosis and Precision Treatment, Xiamen Key Laboratory of Brain Center, the First Affiliated Hospital of Xiamen University, School of Medicine, Xiamen University, Xiamen, 361102 China; 2 The School of Clinical Medicine, Fujian Medical University, Fuzhou, 350122 China; 3 Department of Medical Oncology, the First Affiliated Hospital of Xiamen University, Xiamen, 361003 China

**Keywords:** CNS cancer, Double-strand DNA breaks

## Abstract

Temozolomide (TMZ) is widely utilized as the primary chemotherapeutic intervention for glioblastoma. However, the clinical use of TMZ is limited by its various side effects and resistance to chemotherapy. The present study revealed the synergistic inhibition of glioblastoma through the combined administration of TMZ and perifosine. This combination therapy markedly diminished BRCA1 expression, resulting in the suppression of DNA repair mechanisms. Furthermore, the combination of TMZ and perifosine elicited caspase-dependent apoptosis, decreasing glioblastoma cell viability and proliferation. The observed synergistic effect of this combination therapy on glioblastoma was validated in vivo, as evidenced by the substantial reduction in glioblastoma xenograft growth following combined treatment with TMZ and perifosine. In recurrent glioma patients, higher BRCA1 expression is associated with worse prognosis, especially the ones that received TMZ-treated. These findings underscore the potent antitumor activity of the AKT inhibitor perifosine when combined with TMZ and suggest that this approach is a promising strategy for clinical glioblastoma treatment.

## Introduction

Glioblastoma is the most prevalent brain tumor and is characterized by high aggressiveness, a high recurrence rate, and heterogeneity [1–3]. The primary clinical treatment is surgical resection followed by adjuvant radiotherapy and chemotherapy (temozolomide, TMZ) [4]. Considering the detrimental side effects of TMZ and the issue of chemotherapy resistance, the existing multimodal therapies are inadequate for improving the outcomes of patients with glioblastoma [5, 6]. Therefore, effective drugs with low toxicity are urgently needed for glioblastoma treatment.

The tumor suppressor breast cancer type 1 susceptibility protein (BRCA1) plays an essential role in repairing double-strand DNA breaks through homologous recombination, helping to maintain genomic stability [7]. The role of BRCA1 mutations varies in different tumors. For example, BRCA1 mutations are associated with longer survival in ovarian and endometrial cancers, but shorter survival in breast cancer [8]. In tamoxifen-resistant breast cancer, the activated PI3K/AKT pathway is responsible for BRCA1 upregulation [9]. AKT inhibitor treatment of tumor-bearing BRCA1-mutant mice significantly reduced tumor volume [10]. BRCA1, traditionally considered a tumor suppressor, plays an unexpected tumor-promoting role in glioblastoma [11]. Recent studies have found that higher levels of BRCA1 expression are associated with lower survival in low-grade gliomas [12]. As a key protein of the FA pathway, BRCA1 is involved in DNA damage repair induced by DNA alkylating agents [5]. BKM120 reduces BRCA1 expression, affects the ability to repair DNA damage, and therefore increases PARP inhibitor-induced cell death [13].

PI3K/AKT/mTOR signaling is activated in various cancers, including glioblastoma, and has been correlated with high chemoresistance [14–16]. Previous studies suggest that AKT is extensively activated and highly correlated with malignancy and tumor progression in glioma [17, 18]. Therefore, AKT is an attractive therapeutic target. Perifosine was the first identified allosteric AKT inhibitor and has been found to be able to penetrate the blood‒brain barrier in nonhuman primate and mouse models [19, 20]. Perifosine inhibits AKT by targeting its pleckstrin homology domain, thus preventing its translocation to the plasma membrane and subsequent phosphorylation [21]. A phase I clinical trials of perifosine have indicated that perifosine is safe and tolerable in both children and adults [22, 23]. A phase II open-label single-arm clinical trial of perifosine revealed radiographic evidence of improvements in approximately 20% of patients with high-grade glioma, with one confirmed partial response [24]. However, among all 16 patients with recurrent glioblastoma, the median progression-free survival (PFS) was 1.58 months, and the median overall survival (OS) was 3.68 months [24]. Bevacizumab, an FDA-approved drug for the treatment of recurrent glioblastoma, had PFS and OS of 3.8 and 9.7 months, respectively [25, 26]. It is clear that perifosine is ineffective in the treatment of recurrent glioblastoma compared to bevacizumab. Even though perifosine is ineffective as a monotherapy for glioblastoma, it was found to enhance bevacizumab-induced apoptosis and the therapeutic efficacy of bevacizumab in a heterotopic glioblastoma model [27]. Additionally, TMZ is the primary drug used for glioblastoma treatment, and the challenges of drug resistance and side effects significantly impact its therapeutic efficacy. However, the potential utility of administering perifosine in combination with TMZ to increase sensitivity to TMZ has not been determined.

In our study, we found that the combination of TMZ and perifosine synergistically inhibited glioblastoma. This combination therapy significantly decreased BRCA1 expression, leading to the inhibition of DNA repair. Additionally, the combination of TMZ and perifosine triggered caspase-dependent apoptosis and decreased glioblastoma cell viability and proliferation. This synergistic therapeutic effect on glioblastoma was further confirmed in vivo by the sharp reduction in glioblastoma xenograft growth after combination therapy with TMZ and perifosine. Our discovery highlights the antitumor activity of the AKT inhibitor perifosine in combination with TMZ, revealing a potential strategy for clinical glioblastoma treatment.

## Results

### Prognostic analysis of TMZ treatment with AKT expression

**Fig. 1:**
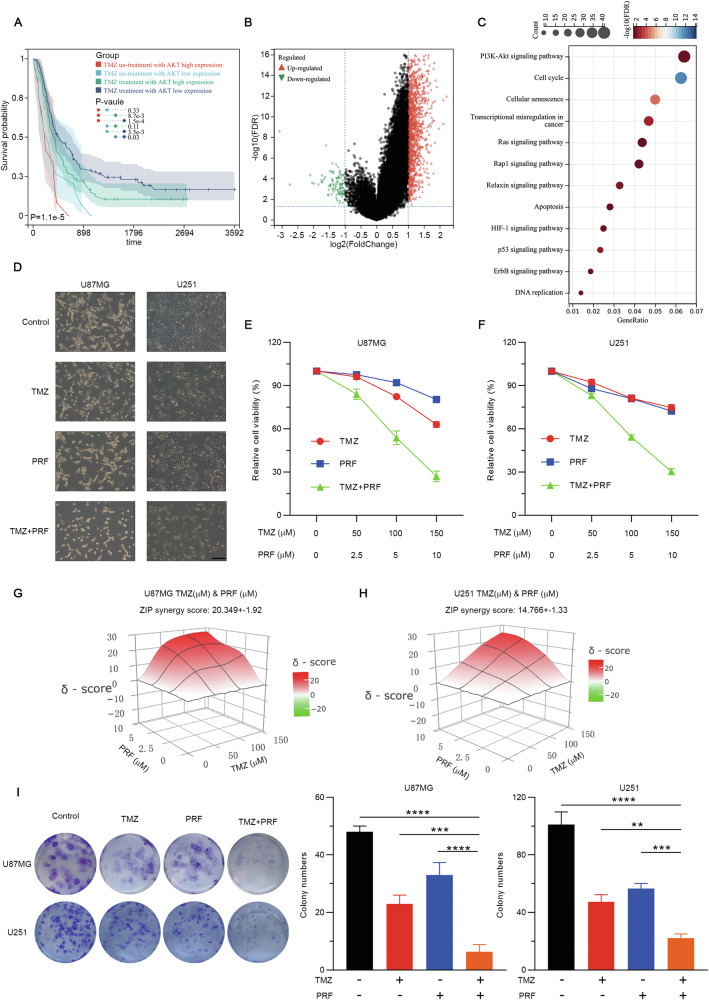
Effects of TMZ and perifosine as monotherapies and a combination therapy on the viability and proliferation of glioblastoma cells. **A** Prognostic analysis of the four groups: non-TMZ-treated + high AKT1 expression, non-TMZ-treated + low AKT1 expression, TMZ-treated + high ATK1 expression, and TMZ-treated + low AKT1 expression. **B** DEGs between the TMZ-treated + high ATK1 expression and TMZ-treated + low AKT1 expression groups. **C** KEGG pathway analysis suggested that apoptosis is the main form of cell death. **D** U87MG and U251 cells were treated with TMZ alone, perifosine (PRF) alone, or TMZ and perifosine in combination for 48 h. The cells were then observed under an inverted microscope. Scale bar: 100 μM. **E**, **F** U87MG and U251 cell viability was determined using a CCK-8 assay after treatment with TMZ (0, 50, 100, 150 μM) alone, perifosine (0, 2.5, 5, 10 μM) alone or TMZ and perifosine in combination for 48 h. **G**, **H** 3D synergy plot of U87MG and U251 cells treated with TMZ alone or in combination with each of the other drugs. The red areas represent synergistic combinations (those with a synergy score of greater than 10). ZIP synergy scores were calculated using the SynergyFinder web application (version 3.0). **I** Colony formation assays were performed to verify the effect of the combination of TMZ and perifosine on glioblastoma cell proliferation. **p < 0.01, ***p < 0.001, and ****p < 0.0001 based on one-way ANOVA followed by Dunnett’s test for multiple comparisons.

The CGGA comprises two datasets (mRNAseq_325 and mRNAseq_693). Prognostic analysis based on TMZ treatment combined with stratification by AKT expression revealed significant differences in prognosis between the group with high AKT expression treated with TMZ and the group with low AKT expression treated with TMZ (Fig. 1A). These findings indicate that elevated AKT expression is associated with poor prognosis in glioblastoma patients treated with TMZ. A total of 1386 DEGs were identified between the two groups: 1292 were upregulated, and 94 were downregulated (Fig. 1B). KEGG pathway enrichment analysis revealed that apoptosis is a prominent mode of cell death (Fig. 1C).

### Synergistic inhibitory effect of combination therapy with TMZ and perifosine on glioblastoma

Perifosine is a pioneering allosteric AKT inhibitor in clinical development that shows good safety and notable penetration across the blood‒brain barrier [19, 22]. Consequently, we opted to use the combination of TMZ and perifosine and conduct a preclinical investigation of their collective therapeutic effect on glioblastoma. Initially, we exposed U87MG and U251 cells to different concentrations of TMZ alone, perifosine alone, or TMZ and perifosine in combination for 48 h (Fig. 1D). The results of CCK-8 assays revealed that TMZ and perifosine monotherapy decreased glioblastoma cell viability in a concentration-dependent manner (Fig. 1E, F). More importantly, combined treatment with TMZ and perifosine markedly decreased the viability of glioblastoma cells, with effects surpassing those observed in the groups treated with either drug alone. To evaluate the potential synergistic anticancer effect of TMZ and perifosine, a quantitative assessment was performed using the synergy score. The dose‒response matrix for patients treated with the combination of TMZ and perifosine for 48 h was uploaded to SynergyFinder (version 3.0). The ZIP synergy scores for the combined treatment in U87MG and U251 cells were 20.349 ± 1.92 and 14.766 ± 1.33, respectively (Fig. 1G, H). These findings indicate the synergistic effect of TMZ and perifosine, with ZIP synergy scores of greater than 10, highlighting the efficacy of the combination of these two agents in inhibiting glioblastoma cell growth after 48 h of treatment. Furthermore, we performed plate colony formation assays to assess the effect of combination therapy with TMZ and perifosine on glioblastoma cell proliferation. As illustrated in Fig. 1I, the number of clones following combined treatment with TMZ and perifosine was significantly lower than that in the groups treated with TMZ or perifosine alone. In conclusion, combined treatment with TMZ and perifosine synergistically decreased glioblastoma cell viability and proliferation.

### Combined treatment with TMZ and perifosine decreases BRCA1 expression to inhibit DNA repair and induce DNA damage

**Fig. 2:**
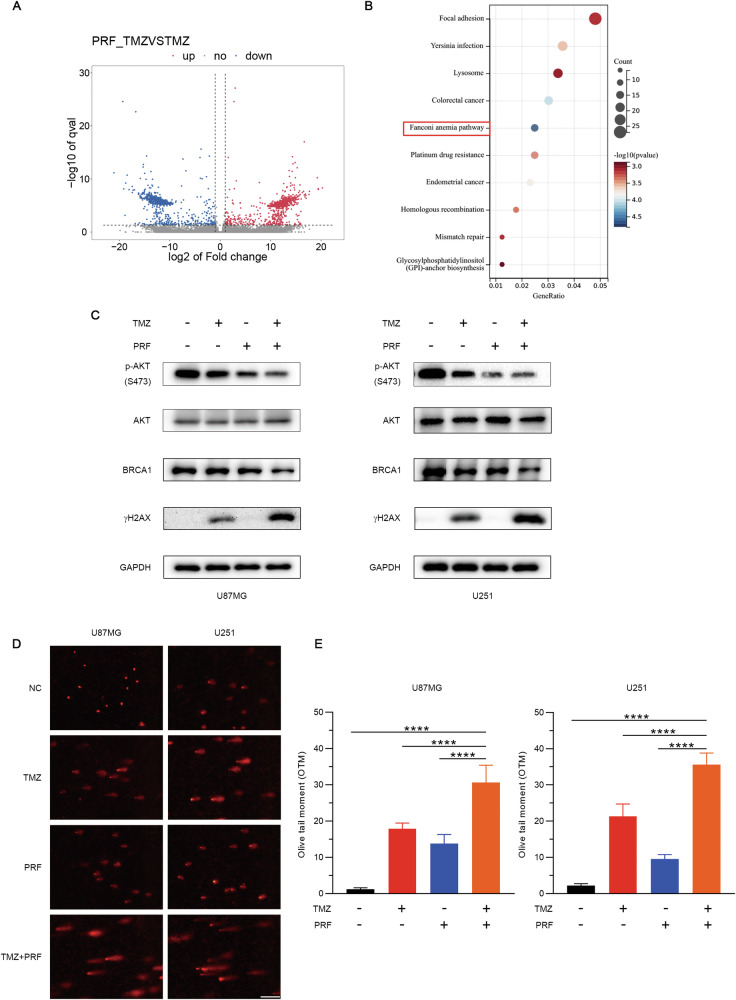
Combined treatment with TMZ and perifosine inhibits DNA repair and induces DNA damage in glioblastoma cells. **A** Volcano plot of the DEGs between the perifosine treatment group and the TMZ/perifosine combination treatment group (upregulated genes are indicated in red; downregulated genes are indicated in blue). **B** The Fanconi anemia pathway was the most significantly enriched pathway among the top 10 signaling pathways. In the scatter plot, the size of a bubble represents the number of genes, and the color of the bubble represents the *q* value determined by enrichment analysis. **C** Western blot analysis confirmed that combined treatment with TMZ and perifosine decreased BRCA1 expression to inhibit DNA repair. **D** DNA damage in glioblastoma cells was evaluated by an alkaline comet assay. Scale bar: 100 μM. **E** The Olive tail moment was calculated with CASP software to quantify DNA damage. **** *p* < 0.0001 based on one-way ANOVA followed by Dunnett’s test for multiple comparisons.

The synergistic effect of TMZ and perifosine resulted in a notable increase in the efficacy of glioblastoma treatment. Therefore, we aimed to explore the detailed mechanisms underlying the synergistic effects of these two chemicals on inhibiting glioblastoma progression. We performed transcriptome analysis of U87MG cells after treatment with perifosine alone or in combination with TMZ. The results of KEGG pathway analysis revealed that the Fanconi anemia pathway was the pathway most strongly affected by the combined treatment (Fig. 2A, B). We further validated the RNA sequencing results at the protein level. Western blot analysis of AKT pathway proteins revealed that the inhibition of AKT by combined treatment with perifosine and TMZ significantly decreased BRCA1 expression to inhibit DNA repair (Fig. 2C). Subsequently, we examined whether this decreased repair capacity results in DNA double-strand breaks and sustained DNA damage signaling. We found that the γH2AX level increased sharply when the cells were further treated with the combination of TMZ and perifosine (Fig. 2C). Moreover, we performed an alkaline comet assay to quantify DNA damage. The results revealed that combined treatment with TMZ and perifosine led to a significant increase in the Olive tail moment in glioblastoma cells (Fig. 2D, E). Overall, these findings indicate that treatment with the combination of TMZ and perifosine decreases BRCA1 expression to inhibit DNA repair and induce DNA damage.

### Combined treatment with TMZ and perifosine induces apoptosis via a caspase-dependent mechanism

**Fig. 3:**
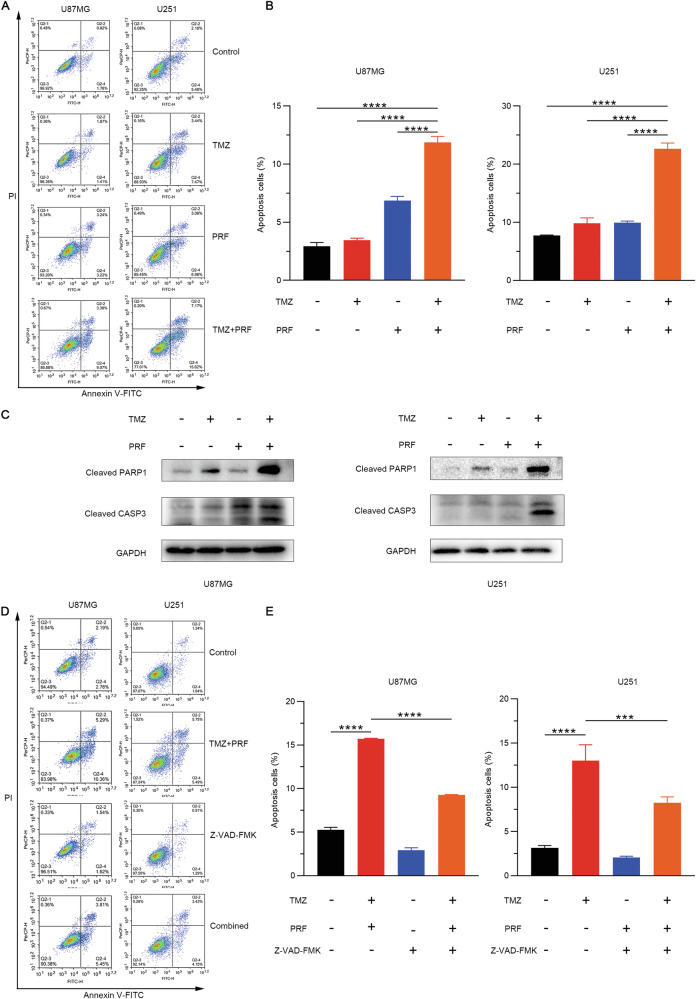
Combined treatment with TMZ and perifosine promotes apoptosis in glioblastoma cells. **A** After U87MG and U251 cells were treated with TMZ alone, perifosine alone, or TMZ and perifosine in combination for 48 h, the percentage of apoptotic cells was determined via flow cytometry using PI/Annexin V-FITC double staining. **B** Histograms illustrating the number and distribution of apoptotic cells in the total cell population. ^****^p < 0.0001 as determined by one-way ANOVA followed by Dunnett’s test for multiple comparisons. **C** After 48 h of treatment with TMZ alone, perifosine alone, or TMZ and perifosine in combination, the expression of proteins associated with apoptosis in U87MG and U251 cells was measured by Western blotting, with GAPDH serving as a loading control. **D** U87MG and U251 cells were treated with the combination of TMZ and perifosine following a 2-h pretreatment with zVAD-FMK, and the percentage of apoptotic cells was determined via flow cytometry using PI/Annexin V-FITC double staining. **E** Histograms illustrating the number and distribution of apoptotic cells in the total cell population. ***p < 0.001, ****p < 0.0001 as determined by Student’s *t* test.

The percentage of apoptotic cells was assessed using an Annexin V-FITC/PI double staining assay. As shown in Fig. 3A, B, combination therapy with TMZ and perifosine significantly increased the percentage of apoptotic U87MG and U251 cells. Furthermore, the levels of the key apoptosis-associated proteins cleaved PARP1 and cleaved caspase-3 were significantly increased in cells treated with the combination of TMZ and perifosine (Fig. 3C). The above data suggested that treatment with the combination of TMZ and perifosine could induce apoptosis in glioblastoma cells. To further confirm this conclusion, we pretreated cells with the pancaspase inhibitor zVAD-FMK for 2 h and then treated the cells for another 48 h with the combination of TMZ and perifosine. The results revealed that zVAD-FMK markedly reduced the number of cells undergoing apoptosis induced by the combined treatment (Fig. 3D, E). In summary, these results indicate that combined treatment with TMZ and perifosine induces caspase-dependent apoptosis in glioblastoma cells.

### Combined treatment with TMZ and perifosine retards xenograft tumor growth in vivo

**Fig. 4:**
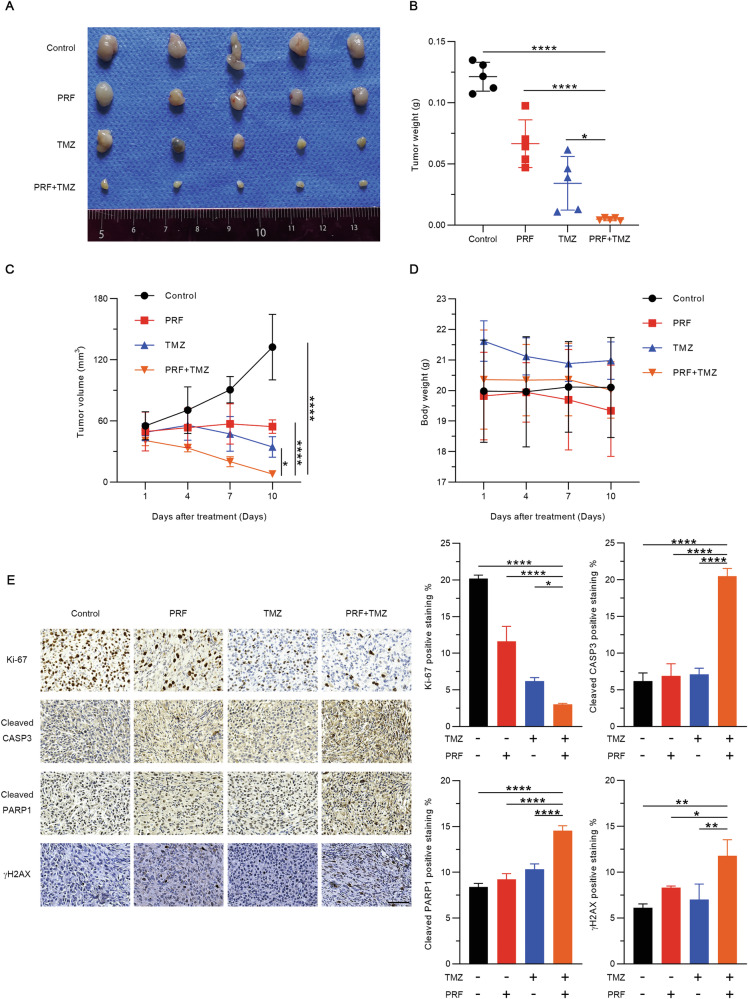
Combination treatment with TMZ and perifosine suppresses glioblastoma xenograft tumor growth in mice. **A** Tumors were excised and photographed at the end of the treatment period. **B** Tumors were excised and weighed at the end of the treatment period. *p < 0.05, ****p < 0.0001 based on one-way ANOVA followed by Dunnett’s test for multiple comparisons. **C** Tumors were measured and tumor volumes calculated every 3 days. **D** The body weights of the glioblastoma xenograft mice were measured every 3 days during the treatment period. **E** Representative immunohistochemical staining for Ki67, cleaved caspase-3, cleaved PARP1, and γ-H2AX in tumor tissues. Scale bar: 100 μM. *p < 0.05, **p < 0.01, ****p < 0.0001 based on one-way ANOVA followed by Dunnett’s test for multiple comparisons.

To validate the synergistic effect of TMZ and perifosine in vivo, we established a subcutaneous xenograft model with glioblastoma cells. The mice were euthanized after a 10-day treatment period with TMZ alone, perifosine alone, or TMZ and perifosine in combination. The xenograft tumors were resected and weighed. The weight and volume of the xenograft tumors after combination treatment with TMZ and perifosine were significantly lower than those after treatment with either TMZ or perifosine alone (Fig. 4A–C). A treatment regimen was considered safer in mice if there was no change in the mouse body weight following treatment (Fig. 4D). We performed immunohistochemical staining to analyze the abundance of Ki-67, cleaved PARP1, cleaved caspase-3, and γ-H2AX. The results revealed a decrease in Ki-67 and increases in cleaved PARP1, cleaved caspase-3, and γ-H2AX in xenograft tumors upon combined treatment with TMZ and perifosine, consistent with the in vitro results (Fig. 4E). In conclusion, combination treatment with TMZ and perifosine retards glioblastoma growth and is thus a potential strategy for clinical glioblastoma treatment.

### High BRCA1 expression is related to TMZ resistance in glioma patients

**Fig. 5:**
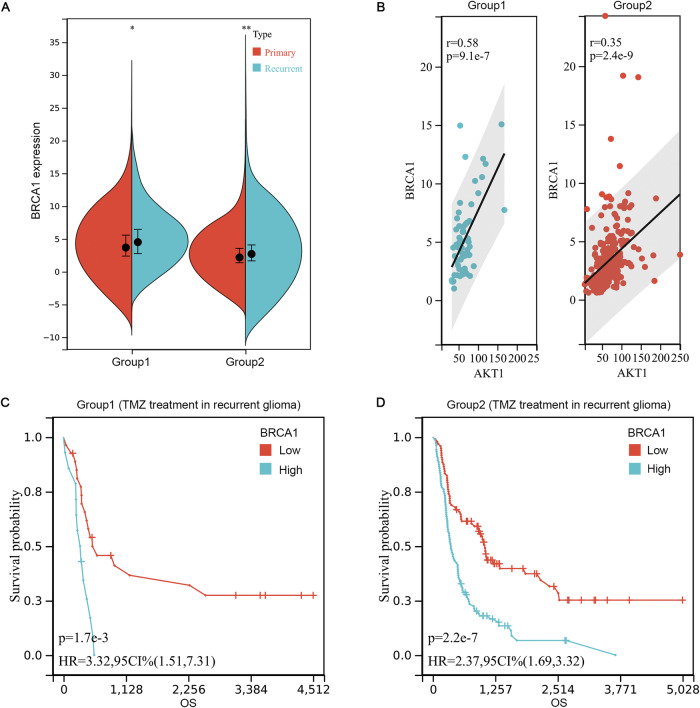
BRCA1 expression is related to TMZ resistance in glioma patients. **A** Expression of BRCA1 in primary and recurrent gliomas. *p < 0.05 and **p < 0.01 compared with primary glioma by Student’s *t* test. **B** Correlation analysis of AKT expression and BRCA1 expression in the indicated recurrent glioma datasets. **C**, **D** OS of recurrent glioma patients treated with TMZ based on BRCA1 expression. (Group 1: mRNAseq_325 datasets; Group 2: mRNAseq_693 datasets).

BRCA1 is involved in DNA damage repair through the FA pathway. Activation of DNA repair pathways is the main mechanism of TMZ resistance in glioma patients[5]. To explore the relationship between BRCA1 and TMZ resistance, we analyzed clinical and bioinformatics data from patients with recurrent glioma in the CGGA database. Analysis of the two datasets suggested that the expression of BRCA1 in recurrent glioma was markedly greater than that in primary glioma (Fig. 5A). TMZ is the drug of choice for the treatment of glioma, and the recurrence of glioma during TMZ treatment indicates the occurrence of TMZ resistance. Therefore, we performed a survival analysis of TMZ-treated patients in recurrent gliomas. The results showed that BRCA1 expression level was inversely correlated with OS in two datasets (Fig. 5C, D). Perifosine is an AKT inhibitor. The expression of BRCA1 in recurrent glioma was positively correlated with AKT (Fig. 5B). Our western blot results also demonstrate that combined treatment with TMZ and perifosine decreases the expression of BRCA1. These data indicate that BRCA1 is a potential target for attenuating TMZ resistance in clinical glioma treatment. The combination of TMZ and perifosine may be a promising strategy to solve this problem.

## Discussion

Glioblastoma is the primary brain tumor of the central nervous system and has a median survival of less than 15 months [28, 29]. Despite its approval by the Food and Drug Administration (FDA) in 2005 as a first-line treatment for glioblastoma, TMZ has exhibited limited efficacy, due primarily to inherent tumor heterogeneity and the rapid emergence of resistance [30, 31]. To improve therapeutic efficacy in patients with glioblastoma, the exploration of new drugs or innovative combinations is imperative to prevent glioblastoma and mitigate the development of TMZ resistance.

**Fig. 6:**
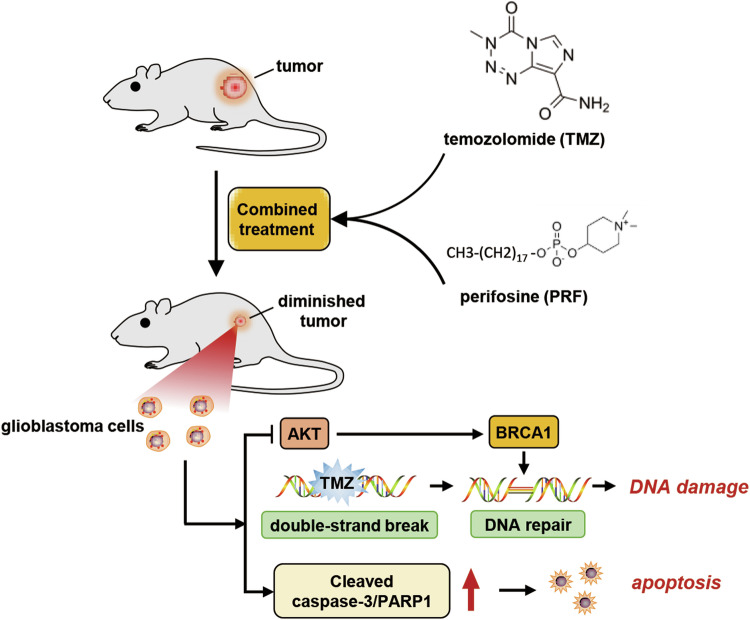
Schematic model of the main results of this study.

In this work, we found that the combination of TMZ and perifosine synergistically inhibited glioblastoma growth. Perifosine, a novel antitumor drug targeting AKT, is currently undergoing clinical trials and premarket development for various malignancies, including leukemia, lung cancer, and colorectal cancer [23, 32, 33]. Previous studies have reported the favorable safety and tolerability of perifosine, as well as its ability to penetrate the blood‒brain barrier. Therefore, we sought to investigate whether perifosine improves the therapeutic effect of TMZ in glioblastoma treatment. First, the synergy score calculated using the SynergyFinder web application was greater than 10, indicating that the combination of perifosine and TMZ exhibited synergistic effects on glioblastoma. Subsequently, we performed RNA sequencing and verified the decrease in BRCA1 expression induced by combined treatment with TMZ and perifosine. The decrease in the level of BRCA1 after combined treatment leads to inhibition of DNA repair and exacerbation of DNA damage. In addition, we found that treatment with the combination of TMZ and perifosine activates caspases to induce apoptosis. Overall, our findings indicated that combined treatment with TMZ and perifosine synergistically inhibited glioblastoma growth by impeding DNA damage repair and inducing apoptosis (Fig. 6).

Previous research by Zhu et al. revealed that activation of the PI3K/AKT pathway was responsible for upregulation of BRCA1 and that treatment with PI3K inhibitors led to a decrease in BRCA1 expression in tamoxifen-resistant cells [9]. BRCA1 was also identified as a modulator of TMZ resistance in p53 wild-type glioblastoma [34]. Thus, inhibiting DNA damage repair by downregulating BRCA1 has emerged as an effective strategy for increasing TMZ sensitivity [13, 35]. According to the CGGA database, BRCA1 expression is strongly correlated with poor prognosis in patients with recurrent glioma, particularly those who underwent TMZ treatment. Our study revealed that the AKT inhibitor perifosine decreases BRCA1 expression, potentially reversing TMZ resistance in patients with recurrent glioma. Despite the unsatisfactory results of clinical trials of perifosine monotherapy for recurrent glioblastoma [24], our preclinical study indicates a promising outlook for future clinical trials exploring the combination of perifosine and TMZ for the treatment of patients with glioma, especially those with a poor prognosis and recurrence. In conclusion, the results obtained in our study suggest that the combination of TMZ and perifosine is a promising strategy for glioblastoma treatment.

## Materials and methods

### Cell culture and reagents

Glioblastoma cell lines (U87MG and U251) were obtained from the American Type Culture Collection (ATCC). The cells were cultured at 37 °C in Dulbecco’s modified Eagle’s medium (DMEM; HyClone, Logan, UT, USA) supplemented with 10% heat-inactivated fetal bovine serum (FBS; ABW; Shanghai, China) and 1% penicillin/streptomycin (Gibco, Carlsbad, USA) in a humidified incubator containing 5% CO_2_.

Perifosine (GC15680) and TMZ (T127425) were purchased from GlpBio and Aladdin, respectively. The pancaspase inhibitor zVAD-FMK (T7020) was purchased from TargetMol. Antibodies specific for cleaved caspase-3, cleaved PARP1, γ-H2AX, and p-AKT (S473) were purchased from Cell Signaling Technology. Antibodies specific for GAPDH, Ki-67, and AKT were purchased from Proteintech. An antibody specific for BRCA1 was purchased from ABclonal.

### Cell viability assay and synergy analysis

U87MG and U251 cells were seeded in 96-well plates at a density of 8,000 cells per well. After 24 h, the cells were treated with perifosine (2.5, 5, or 10 µM) and/or TMZ (50, 100, or 150 µM) for 48 h. Then, 10 μL of Cell Counting Kit-8 (CCK-8) reagent (K1018, ApexBio, Houston, TX, USA) was added to each well. Following 1 h incubation, the optical density (OD) of each well at 450 nm was measured by a microplate reader. Analysis of dose‒response matrix data and calculation of zero interaction potency (ZIP) synergy scores were performed with SynergyFinder. A ZIP synergy score greater than 10 indicates that the interaction between two drugs is likely to be synergistic [36–38].

### Colony formation assay

U87MG and U251 cells were seeded into 6-well plates at a density of 800 cells per well. After 24 h, the cells were treated with TMZ and/or perifosine or with vehicle control. After 14 days, the cells were fixed with 4% paraformaldehyde and stained with 0.5% crystal violet for 20 min. Images were acquired after the cells were rinsed with sterile water. Then, the colonies were counted.

### RNA sequencing

U87MG cells were incubated with TMZ (150 µM) and/or perifosine (10 µM) for 48 h and lysed with TRIzol reagent. The total RNA quantity and purity were assessed using a Bioanalyzer 2100 and a RNA 6000 Nano LabChip Kit (Agilent, CA, USA; 5067-1511), and high-quality RNA samples with an RNA integrity number (RIN) of > 7.0 were used to construct the sequencing library. Then, we performed 2×150 bp paired-end (PE150) sequencing on an Illumina NovaSeq™ 6000 system following the vendor’s recommended protocol.

### Apoptosis assay

Apoptosis in U87MG and U251 cells was analyzed using a PI/Annexin V-FITC kit (556547, BD Biosciences, USA). After 48 h of treatment with TMZ and/or perifosine, cells were trypsinized, collected, and rinsed with precooled PBS. Then, 100 μL of 1×binding buffer containing PI and Annexin V-FITC was added to the cells for 15 min of staining. Subsequently, 400 μL of 1×binding buffer was added, and the percentage of apoptotic cells was determined via flow cytometry.

### Alkaline comet assay

U87MG and U251 cells were seeded into 6-well plates (1.5 × 10^5^ cells per well). After 24 h, the cells were treated with TMZ and/or perifosine for another 48 h. DNA damage was evaluated with a comet assay kit (C2041M; Beyotime, Shanghai, China) according to the manufacturer’s instructions. The Olive tail moment (OTM) was calculated using the Comet Assay Software Project (CASP).

### Western blot analysis

Cells were cultured in a 60 mm dish with vehicle, TMZ (150 µM) alone, perifosine (10 µM) alone, or the combination of TMZ and perifosine. After 48 h, the medium was removed, and the cells were washed twice with precooled PBS. Then, the cells were lysed on ice for 30 min with RIPA lysis buffer supplemented with phenylmethanesulfonyl fluoride (PMSF) and protease/phosphatase inhibitor cocktails and were then centrifuged at 15,000 rpm for 15 min. The supernatant was collected, and the protein concentration was determined by the BCA method. Subsequently, SDS loading buffer was added to the lysate, which was subsequently boiled at 100 °C for 5 min. Equivalent amounts of protein were loaded onto a gel. The proteins were separated by SDS‒PAGE and then transferred to a PVDF membrane (Millipore, Billerica, MA). After 1 h of blocking with 5% nonfat milk, the membrane was incubated with the corresponding primary antibody on a shaker at 4 °C overnight. The PVDF membrane was then incubated with HRP-conjugated goat anti-mouse or anti-rabbit secondary antibody for 1 h at room temperature. Proteins were visualized by enhanced chemiluminescence (ECL) (P10300, NCM Biotech, Suzhou, China) using a chemiluminescence imager.

### Subcutaneous xenograft model

Female nude mice (BALB/c nude, 5–6 weeks old, 18–20 g) were obtained from the Xiamen University Animal Experiment Center and housed at the animal barrier facility of Xiamen University School of Medicine. After one week of acclimatization, U87MG cells (5 × 10^6^ cells per mouse) were injected subcutaneously into the nude mice. The tumors were allowed to grow to a maximal volume of 50 mm^3^, and the animals were subsequently randomly divided into four groups. The mice in these groups (five mice per group) were treated with vehicle, perifosine (15 mg/kg, i.p.) alone, TMZ (5 mg/kg, i.p.) alone, or TMZ and perifosine in combination (at the same doses used for the single-agent treatments). The body weights of the nude mice and the volumes of the tumors were measured every 3 days. The tumor volume was calculated using the equation V = L ×W^2^ × 1/2 (V, volume; L, length; W, width). At the end of the 10-day experimental period, the mice were euthanized. The tumors were excised, weighed and placed in 4% paraformaldehyde. All animal experiments were reviewed and approved by the Animal Ethics Committee of Xiamen University.

### Immunohistochemistry

Tumor tissue was isolated and fixed overnight with 4% paraformaldehyde. Then, the paraffin-embedded tissue was sectioned. Immunohistochemical staining was performed as described previously [39]. Images were acquired with an EVOS M7000 microscope (Thermo Fisher Scientific, Waltham, MA, USA).

### Bioinformatics

The glioma dataset was obtained from the Chinese Glioma Genome Atlas (CGGA; http://www.cgga.org.cn/) database. Prognostic analysis based on TMZ treatment in combination with stratification by AKT expression according to the median value was performed. Differentially expressed genes (DEGs) were identified by screening with the criterion |Log|FC||> 1 (limma package). Kyoto Encyclopedia of Genes and Genomes (KEGG) pathway enrichment analysis was performed with the genes with a *p* value of <0.05.

### Statistical analysis

The experimental data were statistically analyzed using GraphPad Prism 8.0 software. The data are presented as the means ± standard deviations (SDs). One-way analysis of variance (ANOVA) was used for comparisons among multiple groups. *p* values are designated as follows: *: *p* < 0.05; **: *p* < 0.01; ***: *p* < 0.001; ****: *p* < 0.0001.

### Supplementary information

Original Data File

## Data Availability

The datasets generated during and/or analysed during the current study are available from the corresponding author on reasonable request.

## References

[1] Khan F, Pang L, Dunterman M, Lesniak MS, Heimberger AB, Chen P. Macrophages and microglia in glioblastoma: heterogeneity, plasticity, and therapy. J Clin Investig. 2023;133:1. 10.1172/JCI163446 PMC9797335 36594466

[2] Bikfalvi A, da Costa CA, Avril T, Barnier JV, Bauchet L, Brisson L (2023). Challenges in glioblastoma research: focus on the tumor microenvironment. Trends cancer.

[3] Tan AC, Ashley DM, López GY, Malinzak M, Friedman HS, Khasraw M (2020). Management of glioblastoma: state of the art and future directions. CA: A Cancer J Clin.

[4] Schaff LR, Mellinghoff IK (2023). Glioblastoma and other primary brain malignancies in adults: a review. JAMA.

[5] Tomar MS, Kumar A, Srivastava C, Shrivastava A (2021). Elucidating the mechanisms of Temozolomide resistance in gliomas and the strategies to overcome the resistance. Biochimica et Biophys Acta Rev Cancer.

[6] Gong L, Yin Y, Chen C, Wan Q, Xia D, Wang M (2022). Characterization of EGFR-reprogrammable temozolomide-resistant cells in a model of glioblastoma. Cell death Discov.

[7] Tarsounas M, Sung P (2020). The antitumorigenic roles of BRCA1-BARD1 in DNA repair and replication. Nat Rev Mol Cell Biol.

[8] Gammall J, Lai AG (2022). Pan-cancer prognostic genetic mutations and clinicopathological factors associated with survival outcomes: a systematic review. NPJ Precis Oncol.

[9] Zhu Y, Liu Y, Zhang C, Chu J, Wu Y, Li Y (2018). Tamoxifen-resistant breast cancer cells are resistant to DNA-damaging chemotherapy because of upregulated BARD1 and BRCA1. Nat Commun.

[10] Baek HJ, Kim SE, Kim JK, Shin DH, Kim TH, Kim KG (2018). Inhibition of AKT suppresses the initiation and progression of BRCA1-associated mammary tumors. Int J Biol Sci.

[11] Rasmussen RD, Gajjar MK, Tuckova L, Jensen KE, Maya-Mendoza A, Holst CB (2016). BRCA1-regulated RRM2 expression protects glioblastoma cells from endogenous replication stress and promotes tumorigenicity. Nat Commun.

[12] Meimand SE, Pour-Rashidi A, Shahrbabak MM, Mohammadi E, Meimand FE, Rezaei N (2022). The prognostication potential of BRCA genes expression in gliomas: a genetic survival analysis study. World Neurosurg.

[13] Zhang S, Peng X, Li X, Liu H, Zhao B, Elkabets M (2021). BKM120 sensitizes glioblastoma to the PARP inhibitor rucaparib by suppressing homologous recombination repair. Cell Death Dis.

[14] He Y, Sun MM, Zhang GG, Yang J, Chen KS, Xu WW (2021). Targeting PI3K/Akt signal transduction for cancer therapy. Signal Transduct Target Ther.

[15] Verdugo E, Puerto I, Medina M (2022). An update on the molecular biology of glioblastoma, with clinical implications and progress in its treatment. Cancer Commun (Lond, Engl).

[16] Wei L, Ma X, Hou Y, Zhao T, Sun R, Qiu C (2023). Verteporfin reverses progestin resistance through YAP/TAZ-PI3K-Akt pathway in endometrial carcinoma. Cell death Discov.

[17] Mure H, Matsuzaki K, Kitazato KT, Mizobuchi Y, Kuwayama K, Kageji T (2010). Akt2 and Akt3 play a pivotal role in malignant gliomas. Neuro-Oncol.

[18] Daisy Precilla S, Biswas I, Kuduvalli SS, Anitha TS (2022). Crosstalk between PI3K/AKT/mTOR and WNT/β-Catenin signaling in GBM - Could combination therapy checkmate the collusion?. Cell Signal.

[19] Cole DE, Lester-McCully CM, Widemann BC, Warren KE (2015). Plasma and cerebrospinal fluid pharmacokinetics of the Akt inhibitor, perifosine, in a non-human primate model. Cancer Chemother Pharmacol.

[20] Taniguchi K, Suzuki T, Okamura T, Kurita A, Nohara G, Ishii S (2021). Perifosine, a bioavailable alkylphospholipid akt inhibitor, exhibits antitumor activity in murine models of cancer brain metastasis through favorable tumor exposure. Front Oncol.

[21] Gills JJ, Dennis PA (2009). Perifosine: update on a novel Akt inhibitor. Curr Oncol Rep..

[22] Kaley TJ, Panageas KS, Pentsova EI, Mellinghoff IK, Nolan C, Gavrilovic I (2020). Phase I clinical trial of temsirolimus and perifosine for recurrent glioblastoma. Ann Clin Transl Neurol.

[23] Toson B, Fortes IS, Roesler R, Andrade SF (2022). Targeting Akt/PKB in pediatric tumors: a review from preclinical to clinical trials. Pharmacol Res.

[24] Kaley TJ, Panageas KS, Mellinghoff IK, Nolan C, Gavrilovic IT, DeAngelis LM (2019). Phase II trial of an AKT inhibitor (perifosine) for recurrent glioblastoma. J neuro-Oncol.

[25] Weller M, van den Bent M, Preusser M, Le Rhun E, Tonn JC, Minniti G (2021). EANO guidelines on the diagnosis and treatment of diffuse gliomas of adulthood. Nat Rev Clin Oncol.

[26] Tsien CI, Pugh SL, Dicker AP, Raizer JJ, Matuszak MM, Lallana EC (2023). NRG Oncology/RTOG1205: a randomized phase II trial of concurrent bevacizumab and reirradiation versus bevacizumab alone as treatment for recurrent glioblastoma. J Clin Oncol: J Am Soc Clin Oncol.

[27] Ramezani S, Vousooghi N, Ramezani Kapourchali F, Joghataei MT (2017). Perifosine enhances bevacizumab-induced apoptosis and therapeutic efficacy by targeting PI3K/AKT pathway in a glioblastoma heterotopic model. Apoptosis.

[28] Stupp R, Hegi ME, Mason WP, van den Bent MJ, Taphoorn MJ, Janzer RC (2009). Effects of radiotherapy with concomitant and adjuvant temozolomide versus radiotherapy alone on survival in glioblastoma in a randomised phase III study: 5-year analysis of the EORTC-NCIC trial. Lancet Oncol.

[29] Chan P, Rich JN, Kay SA (2023). Watching the clock in glioblastoma. Neuro-Oncol.

[30] Cruz JVR, Batista C, Afonso BH, Alexandre-Moreira MS, Dubois LG, Pontes B, et al. Obstacles to glioblastoma treatment two decades after temozolomide. Cancers. 2022;14:13. 10.3390/cancers14133203 PMC9265128 35804976

[31] Jacob F, Salinas RD, Zhang DY, Nguyen PTT, Schnoll JG, Wong SZH (2020). A patient-derived glioblastoma organoid model and biobank recapitulates inter- and intra-tumoral heterogeneity. Cell.

[32] Adamová B, Říhová K, Pokludová J, Beneš P, Šmarda J, Navrátilová J (2023). Synergistic cytotoxicity of perifosine and ABT-737 to colon cancer cells. J Cell Mol Med.

[33] Hasan GM, Hassan MI, Sohal SS, Shamsi A, Alam M (2023). Therapeutic targeting of regulated signaling pathways of non-small cell lung carcinoma. ACS omega.

[34] Ding J, Wu S, Zhang C, Garyali A, Martinez-Ledesma E, Gao F (2019). BRCA1 identified as a modulator of temozolomide resistance in P53 wild-type GBM using a high-throughput shRNA-based synthetic lethality screening. Am J Cancer Res.

[35] Peng X, Zhang S, Wang Y, Zhou Z, Yu Z, Zhong Z (2023). Stellettin B sensitizes glioblastoma to DNA-damaging treatments by suppressing PI3K-mediated homologous recombination repair. Adv Sci (Weinh, Baden -Wurtt, Ger).

[36] Ianevski A, He L, Aittokallio T, Tang J (2020). SynergyFinder: a web application for analyzing drug combination dose-response matrix data. Bioinforma (Oxf, Engl).

[37] Ianevski A, Giri AK, Aittokallio T (2022). SynergyFinder 3.0: an interactive analysis and consensus interpretation of multi-drug synergies across multiple samples. Nucleic Acids Res.

[38] Zheng S, Wang W, Aldahdooh J, Malyutina A, Shadbahr T, Tanoli Z (2022). SynergyFinder plus: toward better interpretation and annotation of drug combination screening datasets. Genomics Proteom Bioinforma.

[39] Zhao W, Zhang L, Zhang Y, Jiang Z, Lu H, Xie Y (2023). The CDK inhibitor AT7519 inhibits human glioblastoma cell growth by inducing apoptosis, pyroptosis and cell cycle arrest. Cell Death Dis.

