# Supplementary figures and images for: The combination of temozolomide and perifosine synergistically inhibit glioblastoma by impeding DNA repair and inducing apoptosis
Source: Cell Death Discov. 2024 Jul 8;10:315. doi: 10.1038/s41420-024-02085-1 (PMC11231210; doi:10.1038/s41420-024-02085-1)

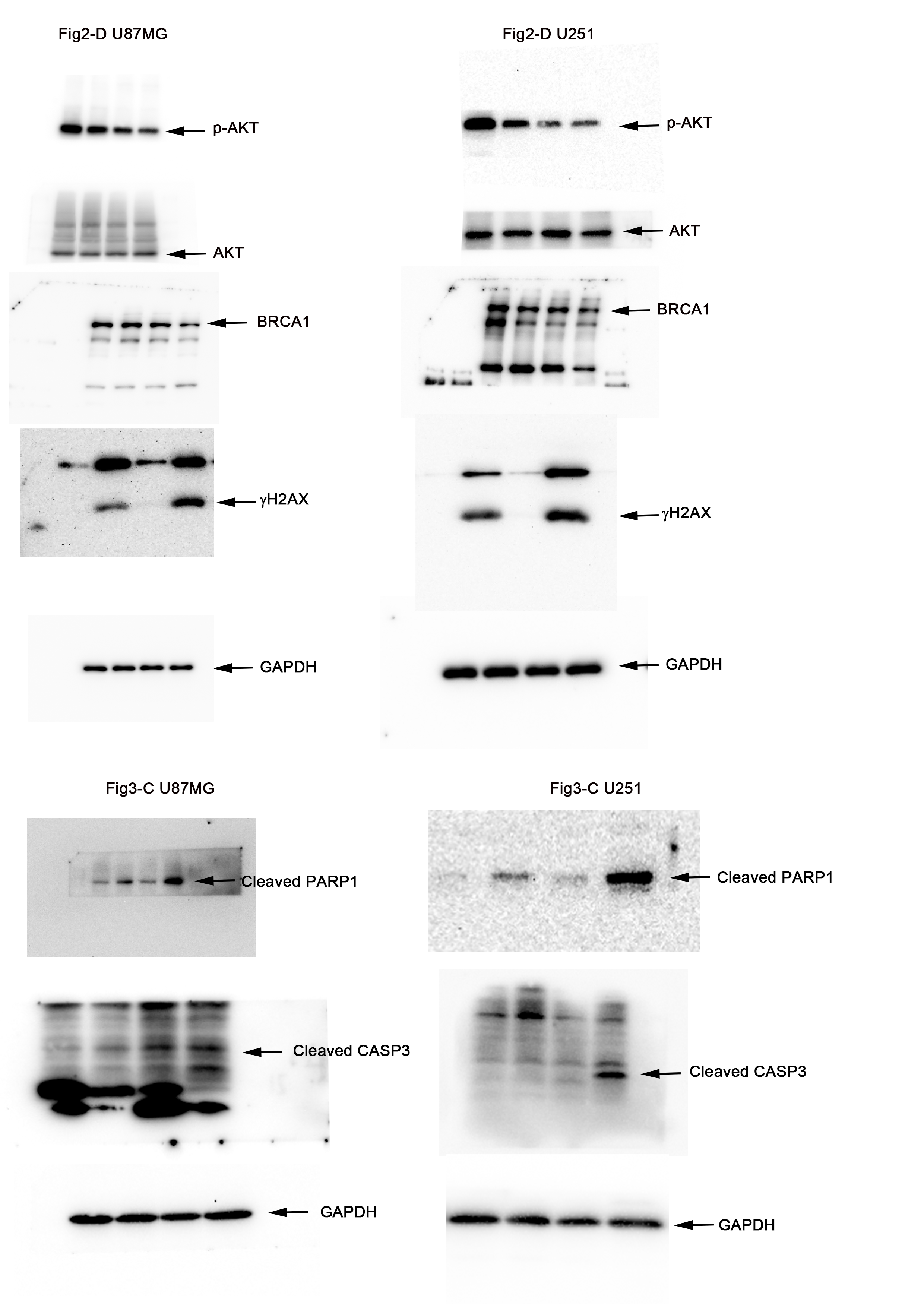

Supplement: Supplementary file 1 — Original Data File [file 41420_2024_2085_MOESM1_ESM.tif]
